# Oral findings and oral health-related quality of life in children with neurofibromatosis type 1: a case-control study

**DOI:** 10.1186/s12903-026-07905-7

**Published:** 2026-02-13

**Authors:** Elif Kabacioglu, Nursel Elcioglu, Murat Can Metin, Funda Kökali, Ali Mentes

**Affiliations:** 1 Department of Pediatric Dentistry, Faculty of Dentistry, Marmara University, Istanbul, Turkey; 2 Department of Pediatric Genetics, Faculty of Medicine, Marmara University, Istanbul, Turkey

**Keywords:** Oral and dental findings, Neurofibromatosis Type1, OHRQoL

## Abstract

**Objective:**

This study aimed to evaluate intraoral findings, oral-health-status, and their relationship with general and oral-health–related-quality-of-life in children diagnosed with neurofibromatosis type1, compared to healthy children.

**Materials and methods:**

Fifty children with neurofibromatosis-type1 and fifty healthy children of similar age and gender underwent thorough oral examinations. Evaluations included the presence of dental caries, enamel hypoplasia, malocclusions, gingiva and plaque levels, and soft tissue abnormalities. Additional assessments included panoramic dental imaging and clinical photographs. General-quality-of-life was measured using a validated child-health-questionnaire, and oral health–related-quality-of-life was assessed through age-appropriate caregiver-reported surveys. Statistical comparisons between groups were conducted using appropriate significance tests, with a threshold of *p* < 0.05.

**Results:**

There were no significant differences between groups in terms of tooth decay, enamel defects, or jaw relationships. Children with neurofibromatosis type 1 had lower gingival health scores and significantly reduced general quality of life. Younger children reported better oral-health–related-quality-of-life, while older children experienced greater family-related impacts.

**Conclusion:**

Although oral health issues in children with neurofibromatosis type 1 were generally mild, their overall and oral health–related quality of life were meaningfully reduced. These findings underscore the importance of including dental professionals in the multidisciplinary care of children with this condition.

**Clinical relevances:**

Recognizing oral signs of NF1 early helps pediatric dentists guide timely referrals and coordinated care. Even mild oral findings can impair children’s quality of life, emphasizing the need for regular monitoring and tailored dental care. Including dentists in multidisciplinary NF1 teams supports both medical and psychosocial well-being.

## Introduction

Neurofibromatosis type 1 (NF1), also known as von Recklinghausen disease, is a neurocutaneous disorder inherited in an autosomal dominant pattern. NF1 results from mutations in the NF1 gene located on chromosome 17q11.2, which encodes the neurofibromin protein [1, 2]. It accounts for approximately 96% of all neurofibromatosis cases, 50% of being familial, 50% sporadic and it affects about 1 in every 3,000 children [3]. No differences in prevalence have been reported based on gender or ethnicity [4]. The disease was first described by Friedrich Daniel von Recklinghausen in 1882 [1], and the diagnostic criteria were established by the U.S. National-Institutes-of-Health (NIH) in 1988 [5].

The primary diagnostic criteria include café-au-lait macules, neurofibromas, Lisch nodules, bone lesions, optic pathway gliomas, and a positive family history [6]. Café-au-lait macules (CALMs), the most prominent clinical feature in children, are observed in nearly all NF1 patients and are often the earliest recognizable sign [7]. Characteristic clinical features appear by the age of one in approximately half of the cases and by the age of eight in 97% of them [8, 9].

Approximately 92% of NF1 patients exhibit findings in the facial, oral, dental, and jaw regions. These include soft tissue neurofibromas, enlargement of fungiform papillae, decreased salivary secretion, and osseous alterations. Dental anomalies such as tooth displacement, impaction, and hypodontia are also commonly observed [10–12]. The most frequently reported oral manifestation in the literature is enlargement of the fungiform papillae of the tongue, observed in approximately half of NF1 patients [12, 13]. Another commonly observed finding is the presence of one or multiple neurofibromas, which most frequently occur on the tongue [12, 14].

Radiographic oral findings in NF1 adult patients may include enlargement of the mandibular canal, mandibular foramen, and mental foramen. Neurofibromas may also develop intraosseously, typically presenting as well-defined, unilocular or, in some cases, multilocular radiolucent lesions. These lesions may lead to tooth impaction [15].

In recent years, oral health has increasingly been recognized as an integral component of overall quality of life. Dental research is no longer limited to treating oral diseases but has also begun to explore the relationship between oral health and quality of life, aiming to improve it [16].

Several validated scales have been developed to assess both general and oral health–related quality of life. For children, the most commonly used tools include the Child Perceptions Questionnaire (CPQ), Child Oral Health Impact Profile (COHIP), Early Childhood Oral Health Impact Scale (ECOHIS), and the Pediatric Oral Health-Related Quality of Life (POQL) scale. In NF1, two key aspects affecting quality of life are the progression and severity of the disease. Although NF1 is typically a slowly progressing condition, it has a significant impact on patients’ quality of life [17].

The aim of this study was to comprehensively evaluate the intraoral findings and oral health status of pediatric patients diagnosed with neurofibromatosis type 1 (NF1), followed at the Departments of Pediatric Genetics and Pediatric Dentistry at Marmara University, and to assess their general health-related and oral health-related quality of life using validated scales (KIDSCREEN-10, POQL, and ECOHIS), comparing these outcomes with those of age- and sex-matched healthy controls.

## Materials and methods

This study was conducted as clinical research at the Department of Pediatric Dentistry, Faculty of Dentistry, Marmara University. Ethical approval was obtained from the Clinical Research Ethics Committee of the Faculty of Dentistry at Marmara University for both the clinical and quality of life components of the study (Number: 2022-93, 12.10.2022). The minimum required sample size was calculated with a confidence level of 95% (1-α), a power of 80% (1-β), and an effect size (d) of 0.6, resulting in 45 participants per group, for a total of 90 participants.

Written informed consent was obtained from the patients and/or their families after they were informed about the scope and purpose of the study. The study included pediatric patients diagnosed with Neurofibromatosis Type 1 based on NIH diagnostic criteria, and who have been followed up at the Department of Pediatric Genetics, Marmara University Faculty of Medicine, Marmara University, since 2010.

Systemic clinical data for the patients were obtained from the archives of the Pediatric Genetics Department. Patients were invited to the Department of Pediatric Dentistry for intraoral examination.

The control group was recruited from healthy children registered at the Department of Pediatric Dentistry, matched by age and gender. A 1:1 case–control ratio was chosen a priori based on efficiency considerations for rare disease studies and feasibility of standardized phenotyping; our sample size computation indicated adequate power to detect the prespecified clinically meaningful differences. The main exclusion criterion for the control group was the presence of any diagnosed or apparent genetic/metabolic disorders.

The following steps were taken in the evaluation of findings in both study and control group children:

- An “Informed Consent Form” was completed after an interview with the parent or accompanying adult.
- A patient information and evaluation form was filled out for each participant. Information provided by the parent or accompanying adult was used to complete the form. In the NF1 group, the following disease-related features were also recorded: presence of café-au-lait macules, freckling, neurofibromas in the head and neck region, facial symmetry, ocular nodules, pseudarthrosis, scoliosis, short stature, and impaired language. The patients were then invited for an intraoral examination at the Department of Pediatric Dentistry.

Intraoral examinations were conducted for all NF1 patients and healthy controls at the Department of Pediatric Dentistry under dental unit light, in a semi-upright position, using a dental mirror and probe, by the same clinician who had undergone standardized training prior to the study to ensure consistency in clinical assessments, between October 2022 and April 2023.

- In each child from both groups, decayed, missing, and filled teeth were assessed using the df-t (for primary teeth) and DMFT (for permanent teeth) indices, and the total score was calculated. The presence or absence of enamel hypoplasia was also evaluated.
- Molar relationships were recorded as Class I, II, or III according to Angle’s classification for permanent dentition, and Baume’s classification for primary dentition. Different types of malocclusions (i.e., open bite, overjet, crossbite, and skeletal anomalies) were also assessed.
- Periodontal tissues were evaluated using the Gingival Index and Plaque Index. The gingival index was recorded using the criteria of Löe and Silness, while the plaque index was recorded using the criteria of Silness and Löe. Each tooth surface was scored between 0 and 3 based on the level of inflammation or plaque accumulation.
- Intraoral and perioral soft tissues were examined via palpation and visual inspection.
- In addition to clinical examinations for caries and dental anomalies, panoramic radiographs were taken based on age and oral findings.
- Intraoral photographs were taken depending on the child’s level of cooperation. Tongue photographs were taken from all patients. The findings were recorded in patient follow-up forms.

For patients in the appropriate age range, the following quality of life questionnaires were completed based on information provided by the parent or accompanying adult:

- Kidscreen-10 Index (Health-Related Quality of Life Questionnaire for Children and Young People and their Parents) [18]
- Pediatric Oral Health-Related Quality of Life (POQL) for children *≥* 8 years of age [19]
- Early Childhood Oral Health Impact Scale (ECOHIS) for children < 8 years of age [20].

All three instruments used in this study (KIDSCREEN-10 Index, POQL, and ECOHIS) are previously developed and validated questionnaires that have been published in the literature, and the corresponding references are cited in this manuscript [18–20].

Participants’ general health-related quality of life (HRQoL) was assessed using the KIDSCREEN-10 Index, a validated 10-item unidimensional questionnaire representing the longer KIDSCREEN instruments. Each item is rated on a five-point Likert scale (1 = never to 5 = always), yielding a total score between 10 and 50, with higher scores indicating better perceived HRQoL [18].

Oral health–related quality of life (OHRQoL) was evaluated using two instruments: the Pediatric Oral Health-Related Quality of Life (POQL) and Early Childhood Oral Health Impact Scale (ECOHIS). The POQL consists of four domains— physical function (2 items), role function (2 items), social impact (3 items) and emotional impact (3 items). Each item is scored according to the frequency of events (ranging from did not happen = 0 to all of the time = 4) and the perceived level of bother, with a total score ranging from 0 to 1. For each item, the frequency score is multiplied by the corresponding bother score, and the resulting values are summed and averaged to generate total and subscale scores; higher scores indicate a greater negative impact of oral health on quality of life [19, 21, 22]. The ECOHIS comprises 13 items, with 9 child impact items and 4 family impact items. Responses are recorded on a five-point Likert scale (0 = never to 4 = very often), yielding a total score between 0 and 52, where higher scores reflect a greater adverse impact on OHRQoL [20, 23].

Oral findings and quality of life scores were compared statistically between the NF1 and control groups. The normality of distribution for numerical variables was tested using the Shapiro-Wilk test. The student’s t-test was used for normally distributed variables, while the Mann-Whitney U test was used for non-normally distributed variables. Relationships between categorical variables were analyzed using the Chi-square test, and multiple comparisons were performed using the Bonferroni test. Analyses were conducted using SPSS version 22.0 for Windows. A p-value of < 0.05 was considered statistically significant.

## Results

A total of 50 pediatric patients diagnosed with NF1 (35 boys and 15 girls) and 50 healthy children (35 boys and 15 girls) were included in the study. The mean age of the NF1 group was 9.36 ± 3.81 years, while the control group’s mean age was 9.34 ± 3.78 years. The youngest patient was 2 years old, and the oldest was 15 years old (*p* = 0.964). Demographic details of both groups are presented in Table 1. In our study, 29 (58%) NF1 patients had a family history of NF1, while 21 (42%) cases were sporadic. Family consanguinity was reported in 24% of the NF1 group and 16% of the control group.

**Table 1:** Demographic data of the NF1 and control groups

		NF1 Group (*n* = 50)	Control Group (*n* = 50)	*p*
Maternal age at pregnancy (year)		29.10 ± 5.51	28.04 ± 5.36	*0.296* ^‡^
Breastfeeding duration (month)		16 ± 14.46	22.14 ± 8.54	0.001^‡^
Bottle feeding duration (month)		13.76 ± 13.74	9.04 ± 11.26	0.071^‡^
Paternal education level	Primary	28 (56%)	23 (46%)	0.534^+^
	Secondary	13 (26%)	14 (28%)	
	University	9 (18%)	13 (26%)	
Maternal education level	Primary	29 (58%)	23 (46%)	0.334^+^
	Secondary	9 (18%)	15 (30%)	
	University	12 (24%)	12 (24%)	
Income level	low	20 (40%)	6 (12%)	0.002^+^
	moderate	27 (54%)	34 (68%)	
	high	3 (6%)	10 (20%)	
Family consanguinity	yes	8 (16%)	12 (24%)	0.317^+^
	no	42 (84%)	38 (76%)	
Medication Use During Pregnancy	yes	6 (12%)	8 (16%)	0.564^+^
	no	44 (88%)	42 (84%)	
Mode of Delivery	Vaginal	22 (44%)	25 (50%)	0.548^+^
	Cesarean	28 (56%)	25 (50%)	
Birth Term	Term	39 (78%)	43 (86%)	0.298^+^
	Preterm	11 (22%)	7 (14%)	
Birth Weight	low weight	7 (14%)	4 (8%)	0.338^+^
	normal	43 (86%)	46 (92%)	
Incubator Stay	yes	10 (20%)	8 (16%)	0.603^+^
	no	40 (80%)	42 (84%)	
Allergy Status	yes	8 (16%)	17 (34%)	0.038^+^
	no	42 (84%)	33 (66%)	
Medication Use	yes	9 (18%)	8 (16%)	0.790^+^
	no	41 (82%)	42 (84%)	
Dental Pain (past 3 Months)	yes	10 (20%)	26 (52%)	0.001^+^
	no	40 (80%)	24 (48%)	
Feeding Difficulties	yes	8 (16%)	13 (26%)	0.220^+^
	no	42 (84%)	37 (74%)	
Toothbrushing Frequency	never	9 (18%)	1 (2%)	0.028^+^
	sometimes	22 (44%)	25 (50%)	
	everyday	19 (38%)	24 (48%)	
Dental Visit (past 3 Months)	yes	9 (18%)	29 (58%)	0.001^+^
	no	41 (82%)	21 (42%)	

‡: Mann Whitney U Test, +: Ki Square Test. A *p* value < 0.05 was considered statistically significant

Systemic and craniofacial findings in children with NF1 are summarized in Table 2. Café-au-lait macules were present in all patients and represented the most common clinical feature. Freckling was also frequently observed, while neurofibromas in the head and neck region were relatively rare. Additional findings included craniofacial asymmetry, Lisch nodules, skeletal abnormalities such as pseudarthrosis and scoliosis, short stature, speech impairment, and learning disabilities. Detailed distributions and percentages of these findings are provided in Table 2.

**Table 2:** Disease-related findings of NF1 patients

Facial Skin manifestations	Café-au-lait macules	Total	42 (84%)
		upper facial region	20 (40%)
		midface region	25 (50%)
		lower facial region	29 (58%)
		neck region	40 (80%)
	Freckling	Total	33 (66%)
		upper facial region	12 (24%)
		midface region	24 (48%)
		lower facial region	18 (36%)
		neck region	29 (58%)
	Neurofibromas		3 (6%)
Craniofacial asymmetry			6(12%)
Ocular manifestations		Lisch nodules	11 (22%)
Skeletal manifestations		Pseudarthrosis	2 (4%)
		Scoliosis	8 (16%)
		Short stature	5 (10%)
Speech impairment			10 (20%)
Learning disability			17 (34%)

Oral findings in the study and control groups are summarized in Table 3. No significant differences were observed between the groups regarding the mean age for the eruption of the first tooth, occlusal relationships, DMFT + dft scores, or enamel hypoplasia. The gingival index was significantly lower in the NF1 group, whereas plaque index values did not differ significantly between groups. Detailed numerical data are provided in Table 3.

**Table 3:** Oral findings of the study and control groups

		NF1 Group (*n* = 50)	Control Group (*n* = 50)	*p*
Eruption of the first tooth (month)		7.22 ± 2.17	7.68 ± 2.57	0.464^‡^
DMFT + dft score		8.76 ± 5.24	10.24 ± 3.55	0.102*
Occlusal relationships	Class I	25 (50%)	18 (36%)	0.322^**+**^
	Class II	10 (20%)	15 (30%)	
	Class III	15 (30%)	17 (34%)	
Enamel hypoplasia		14 (28%)	11 (22%)	0.488^+^
GI	Normal	20 (40%)	11 (22%)	0.033^+^
	Color change	21 (42%)	34 (68%)	
	Inflammation	9 (18%)	5 (10%)	
PI	0	7 (14%)	6 (12%)	0.217^+^
	1	38 (76%)	43 (86%)	
	2	5 (10%)	1 (2%)	

‡: Mann Whitney U Test, *: Student T Test, +: Ki Square Test. A *p* value < 0.05 was considered statistically significant

**Fig. 1:**
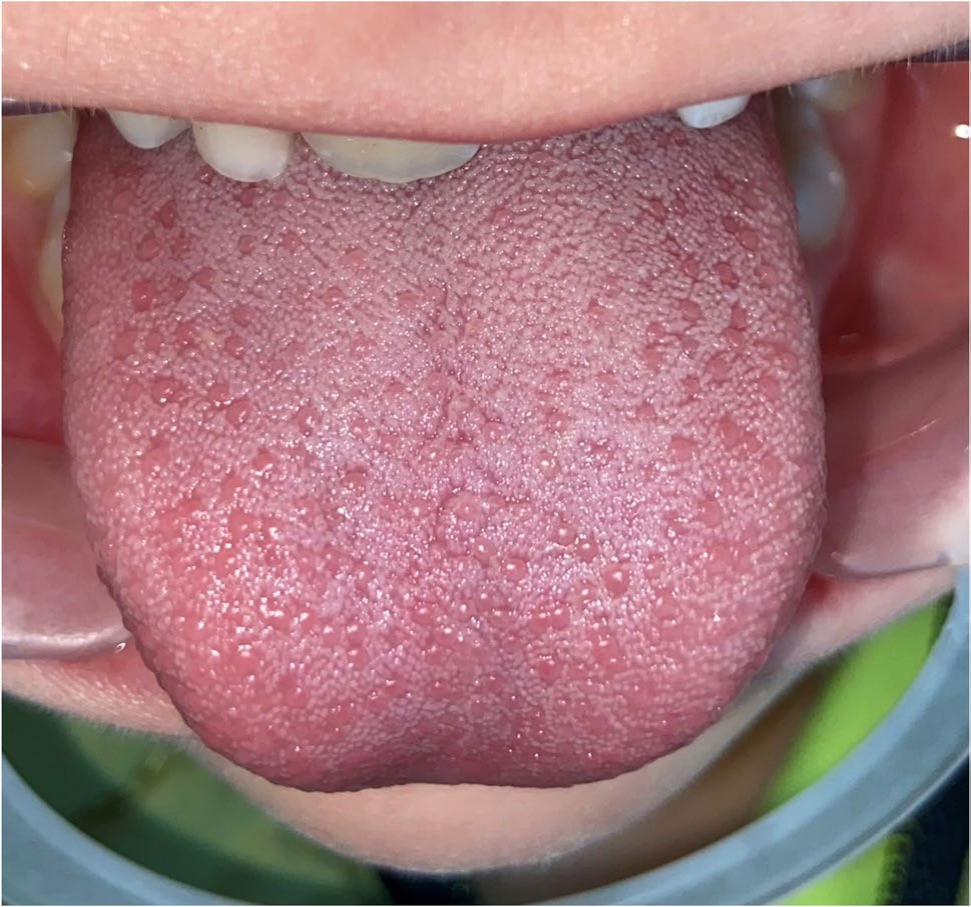
Clinical photograph of papillary hypertrophy on the tongue in a 7-year-old boy with NF1

**Fig. 2:**
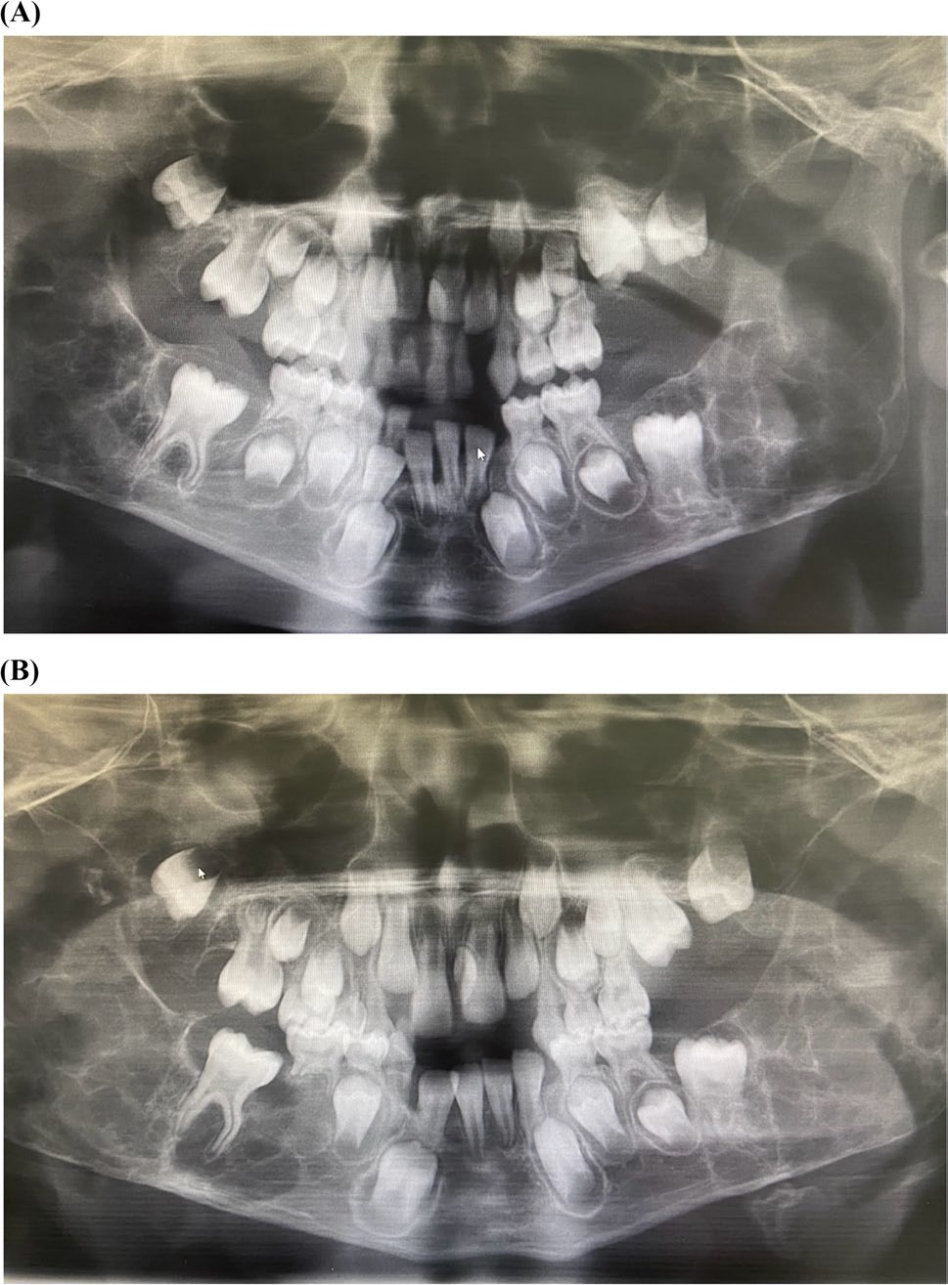
**A** Preoperative panoramic radiograph of bilateral, multilocular osteolytic lesions in a 7-year-old boy with NF1. **B** Postoperative panoramic radiograph of bilateral, multilocular osteolytic lesions in a 7-year-old boy with NF1

Various oral findings in NF1 children were identified, including papillary hyperplasia in 5 patients (Fig. 1), gingival pigmentation in 10 patients, fissured tongue in 2 patients, hypertrophic frenulum in 1 patient, and talon cusp in 1 patient. Based on radiographic evaluation, supernumerary teeth were observed in 4 patients, and tooth agenesis in 3 patients within the NF1 group. In one 7-year-old male patient, a bilateral, multilocular osteolytic lesion causing facial swelling was detected. Histopathological examination revealed that the lesion was consistent with a central giant cell granuloma (Figs. 2). A definitive diagnosis of NF1 was subsequently established following genetic evaluation.

In the KIDSCREEN-10 scale, children with NF1 had lower total scores (35.62 ± 6.26) than the control group (41.56 ± 4.10), and the difference was statistically significant (*p* = 0.001) (Table 4). However, no statistically significant difference was found between groups based on parents’ assessments of their child’s overall health status (*p* = 0.068).

**Table 4:** Comparison of KIDSCREEN-10, POQL and ECOHIS scores between NF1 and control groups

	Variable	NF1 Group Mean ± SD	Control Group Mean ± SD	p-value
KIDSCREEN-10	**Total Score**	35.62 ± 6.26	41.56 ± 4.10	0.001*
POQL	**Physical Function**	0.12 ± 0.16	0.21 ± 0.24	0.177‡
	**Role Function**	0.04 ± 0.07	0.07 ± 0.15	0.929‡
	**Social Impact**	0.09 ± 0.19	0.17 ± 0.29	0.199‡
	**Emotional Impact**	0.06 ± 0.08	0.10 ± 0.14	0.383‡
	**Child Impact Total Score**	0.07 ± 0.09	0.14 ± 0.15	0.161‡
	**Parent Impact Total Score**	0.07 ± 0.11	0.10 ± 0.09	0.041‡
ECOHIS	**Child Impact Total Score**	1.94 ± 2.24	4.81 ± 4.04	0.043‡
	**Family Impact Total Score**	1.50 ± 2.48	4.69 ± 3.46	0.004‡
	**Total Score**	3.44 ± 4.21	9.50 ± 6.41	0.003‡

*: Student T Test, ‡: Mann Whitney U Test. A *p* value < 0.05 was considered statistically significant

The POQL questionnaire was administered to 34 (68%) children in each group (aged *≥* 8). No statistically significant difference was found between the NF1 and control groups based on parents’ assessments (*p* = 0.536). However, a statistically significant difference was found in the mean total score evaluating family impact (*p* = 0.041). In the NF1 group, the physical functioning subscale was found to be the most affected. No significant differences were found in the mean scores of the physical or role functioning, social, or emotional impact subscales (*p* > 0.05) (Table 4).

In children < 8 years of age, the ECOHIS quality of life questionnaire was administered to 16 (32%) children. A statistically significant difference was observed between the groups in the child impact subscale (*p* = 0.043), the family impact subscale (*p* = 0.004), and the total ECOHIS scores (*p* = 0.003) (Table 4).

## Discussion

A thorough understanding of the epidemiology and clinical outcomes of Neurofibromatosis Type 1 (NF1) is essential to providing optimal healthcare and enhancing the quality of life for those affected by the condition. To advance this understanding, increased research and educational efforts are needed to better grasp the complexities of NF1 and improve patient care. Our study addresses these needs by examining the oral manifestations observed in pediatric patients with NF1 and investigating their impact on oral health-related quality of life (OHRQoL), offering valuable insights into this often-overlooked aspect of the condition.

Multiple studies conducted in Turkey have explored the clinical characteristics of pediatric patients diagnosed with NF1. In the study by Çarman et al. [24], involving 49 children, café-au-lait macules were present in all patients, with additional features such as axillary freckling (30.6%), Lisch nodules (40.8%), and neurofibromas (20%). Psychiatric and developmental disorders were noted in 20% of cases. Yılmaz et al. [25] evaluated 183 children and reported a positive family history in 62.8% of cases, café-au-lait spots in all patients, and optic gliomas in 13%. Kaçar et al. [26], in a cohort of 52 patients, observed a high rate of skeletal abnormalities (26.9%) and reported developmental and behavioral problems in 66% of children. Kamış and Küpheli [27] studied 50 patients with confirmed NF1 gene analysis, reporting axillary freckling in 82%, Lisch nodules in 36%, and optic pathway gliomas in 12% of patients. Finally, Eker et al. [28] retrospectively analyzed 157 children presenting with café-au-lait macules and confirmed NF1 diagnosis in 69.4% based on NIH criteria, with optic gliomas in 24.8% and neurofibromas in 17.1% of patients. In our cohort, consisting of pediatric patients diagnosed with NF1, a first-degree family history was identified in 58% of cases. Café-au-lait macules were observed in all patients, with 84% showing lesions specifically in the head and neck region. Similarly, freckling was noted in the head and neck area in 66% of cases. Neurofibromas were identified in 6% and Lisch nodules in 22% of patients, both of which were lower than reported in previous studies. Craniofacial asymmetry was observed in 12% of patients, while skeletal anomalies were present in 20%, most commonly scoliosis (16%) and pseudarthrosis (4%). Short stature (10%), epilepsy (4%), and speech disorders (20%) were also documented. Notably, 34% of patients were enrolled in special education programs due to learning disabilities, reflecting a substantial neurodevelopmental burden.

Several studies have explored the oral manifestations of NF1, particularly focusing on dental development, enamel defects, and occlusal relationships. Lammert et al. [29] reported accelerated eruption of the first tooth in NF1-affected children compared to their unaffected siblings, suggesting a potential influence of the disease on tooth development. However, this observation has not been consistently replicated in subsequent studies, raising the possibility that variations in eruption timing might be influenced by genetic, environmental, or hormonal factors rather than NF1 itself. Similarly, while Santoro et al. [30] documented a significantly higher prevalence of enamel defects and Bardellini et al. [31] and Santoro et al. [30] both noted increased rates of malocclusion, particularly Class III, these findings have not been universally observed. For instance, Jaasaari et al. [32] found no significant differences in tooth development stages between NF1 patients and healthy controls, and some studies have not emphasized enamel or occlusal abnormalities, indicating ongoing debate regarding the consistency of these oral findings in NF1 populations.

In contrast to some of the studies mentioned above, our study did not find significant differences in the timing of first tooth eruption, the number of teeth across different dentition phases, enamel defects, or occlusal relationships between NF1 and control groups. These discrepancies could be attributed to our relatively small, homogenous sample, the cross-sectional design, and potential differences in diagnostic criteria or methods used to assess enamel defects and occlusal status. Additionally, factors such as local oral health practices, fluoride exposure, or socio-economic conditions, which were not assessed in our study, may have contributed to these variations. While our findings align with those of Jaasaari et al. [32] regarding tooth number and development stages, they diverge from the reports of Santoro et al. [30] and Bardellini et al. [31] concerning enamel hypoplasia and malocclusion prevalence. These results highlight the complexity and heterogeneity of oral manifestations in NF1 and emphasize the need for larger, multicenter, prospective studies with standardized diagnostic protocols to clarify these inconsistencies and support the development of tailored dental care approaches for NF1 patients.

Several studies have explored the prevalence of dental caries in patients with NF1, producing mixed results. Shapiro et al. [12] reported a high prevalence of dental caries among both pediatric and adult NF1 patients; however, they suggested that this could be attributed to the generally high caries prevalence in the broader population, rather than being a syndrome-specific feature. Similarly, Tsang et al. [33], Bardellini et al. [31], Santoro et al. [30], and Thota et al. [34] all reported no significant differences in caries prevalence when comparing children with NF1 to healthy controls. In contrast, some studies have reported statistically significant findings. For example, Friedrich and Reul [35] found a higher caries prevalence in adolescents and adults with NF1, whereas Visnapuu et al. [36] reported a lower risk of caries among children and adults with NF1 and, in a later study [37], noted a progressive increase in caries prevalence with age in individuals under 35 years. More recently, Friedrich and Schön [38] observed that caries prevalence was significantly higher in control individuals compared to those with NF1. In light of these varied results, our study aligns with the majority of previous investigations reporting no statistically significant difference in dental caries prevalence between NF1 patients and healthy controls. This supports the suggestion that caries risk in NF1 patients may not differ substantially from the general population, although discrepancies across studies emphasize the possible influence of local factors, oral hygiene habits, and socio-economic status.

Similarly, gingival health outcomes in patients with NF1 have been inconsistently reported in the literature. Shapiro et al. [12] described a high prevalence of periodontal disease in both children and adults with NF1, though they attributed this finding to the overall high incidence of periodontal issues in the general population rather than a direct link to NF1. Bardellini et al. [31] found no significant difference in gingival health, as assessed by CPI scores, between NF1 and control groups. Conversely, Santoro et al. [30] and Thota et al. [34] both reported significantly higher prevalence of gingivitis in pediatric and adult NF1 patients, suggesting a possible predisposition toward gingival inflammation in these populations. Contrasting with these reports, our study found a significantly lower prevalence of gingivitis in the NF1 group compared to healthy controls. This divergence may reflect differences in study design, age groups assessed, or possibly better oral hygiene awareness and care among our NF1 patients, although further studies are needed to confirm these observations.

The literature also presents varying findings regarding oral hygiene status in NF1 patients. Thota et al. [34] reported no statistically significant difference in Plaque Index (PI) scores among NF1 patients under 20 years of age, suggesting that oral hygiene in this age group may not be particularly compromised by the syndrome. In contrast, Bardellini et al. [31] observed significantly poorer oral hygiene, as indicated by lower OHI-S scores in children with NF1, and Santoro et al. [30] similarly found significantly worse oral hygiene conditions among their NF1 pediatric population. In line with the findings of Thota et al. [34], our study also did not detect significant differences in PI scores between NF1 patients and controls. This result suggests that, at least in our sample, NF1 does not appear to impact plaque accumulation or oral hygiene status adversely, though, as with other findings, the role of caregiver involvement, education, and socio-economic background may play important roles that warrant further investigation.

Several studies have documented a high prevalence of oral soft tissue changes in patients with NF1, particularly in adult populations. Visnapuu et al. [37] reported a notably high frequency of oral soft tissue alterations in both children and adults with NF1, underscoring the diverse spectrum of mucosal and gingival manifestations associated with the disorder. Similarly, Shapiro et al. [12] and Santoro et al. [30] described a higher rate of oral neurofibromas among their patient cohorts, suggesting that intraoral neurofibromas, although less common in children, can be present and should be carefully evaluated. In contrast, Bardellini et al. [31], focusing exclusively on pediatric patients, did not observe any oral neurofibromas in their sample. In our study, oral soft tissue changes were observed less frequently than in the aforementioned studies. While we did identify neurofibromas in the head and neck region, no intraoral neurofibromas were observed, which is consistent with the findings of Bardellini et al. [31]. Additionally, papillary hypertrophy of the tongue was noted in our cohort, a finding that aligns partially with Shapiro et al. [12], who reported a higher prevalence of fungiform papilla enlargement, and with Jouhilahti et al. [39], who observed prominent tongue papillae. Furthermore, gingival pigmentation and fissured tongue were documented in our study; however, these specific manifestations have not been extensively reported in prior studies, suggesting that they might represent under-recognized features of the oral phenotype in NF1 patients.

Radiographic evaluations in patients with NF1 have also been the subject of several studies, most of which report various skeletal and dental anomalies. Bardellini et al. [31] documented radiographic abnormalities involving the jaws and temporomandibular joint (TMJ) structures in their pediatric NF1 cohort, while Visnapuu et al. [40] described a wide range of radiologic findings in both children and adults, emphasizing the importance of imaging in comprehensive NF1 assessments. Similarly, Friedrich and Reul [41] reported frequent skeletal anomalies, including alterations of the mandibular ramus, glenoid fossa, condylar deformities, and enlargement of the mandibular foramen, further corroborating the relevance of detailed radiological examination. Although Friedrich and Scheuer [42] observed these anomalies at a lower frequency, their reported rates still exceeded those found in our study. In our cohort, jaw anomalies were detected radiographically in only one patient, indicating a lower frequency compared to the literature, which might be attributed to the limited sample size or the younger age of our study population.

With respect to dental anomalies, our study observed supernumerary teeth in 8% of patients, a finding that is consistent with rates reported in the literature. Friedrich et al. [43] similarly found comparable frequencies of supernumerary teeth in both pediatric and adult NF1 patients. However, earlier studies by the same research group [10, 44] reported lower rates, suggesting a possible increase in recognition of these anomalies in more recent evaluations. In terms of hypodontia, our study identified a prevalence of 6%, which is notably lower than the significantly higher rates of hypodontia reported by Friedrich et al. [10] in their NF1 cohort. This difference may be reflective of variations in sample size, demographic factors, or diagnostic criteria used across studies.

Numerous studies have consistently demonstrated that children and adults with neurofibromatosis type 1 (NF1) experience significantly reduced health-related quality of life (HRQoL) compared to healthy individuals. Saltık and Başgül [45] reported that increased age and greater clinical burden were associated with lower PedsQL scores across all domains. Roy et al. [46] found that disease severity and visibility negatively impacted both parent- and self-reported HRQoL, particularly in emotional, social, and physical domains. Similarly, Yoshida et al. [47] observed lower EQ-5D-5 L and VAS scores in NF1 adults, while Dhaenens et al. [48] reported significantly lower HRQoL in NF1 children aged 5–12, especially in physical health. Cavallo et al. [49] further linked higher severity and visibility scores with decreased physical and social functioning. These findings are in strong concordance with our study, in which KIDSCREEN-10 scores were significantly lower in the NF1 group, confirming a broad and persistent QoL deficit in pediatric NF1 patients. Our results not only reinforce the growing body of evidence that NF1 has a profound negative impact on children’s overall well-being, but also highlight the importance of integrating routine HRQoL assessments into clinical follow-up. Early recognition of these psychosocial and functional challenges may help guide multidisciplinary interventions aimed at improving the quality of life for NF1-affected children and their families.

To the best of our knowledge, the impact of oral health on quality of life in patients with neurofibromatosis type 1 (NF1) has not yet been evaluated using validated instruments, indicating a notable gap in the existing literature. While several studies have extensively documented the general health-related quality of life impairments in NF1 patients, the specific contribution of oral health factors to these deficits has remained unexplored. Our study is, therefore, among the first to systematically evaluate OHRQoL in children with NF1 using validated, age-appropriate instruments such as the Early Childhood Oral Health Impact Scale (ECOHIS) and the Pediatric Oral Health-Related Quality of Life Scale (POQL). Interestingly, we observed that NF1 patients under the age of 7 reported better OHRQoL scores compared to their healthy counterparts, despite similar clinical oral health status. This unexpected finding might be explained by the fact that the control group was drawn from a dental clinic setting, potentially leading to heightened oral health awareness and concern among these families. Conversely, in the older age group (8 years and above), while children’s self-reported OHRQoL did not differ significantly between groups, parents of NF1 children reported a lower perceived family impact from oral health issues compared to controls. This discrepancy between parental and child perceptions suggests that as children age and systemic manifestations of NF1 become more apparent, oral health may become relatively less of a perceived burden compared to other health concerns. Moreover, physical functioning emerged as the most affected domain of overall quality of life in NF1 patients, underscoring the broader impact of the disease beyond the oral cavity. Although our results did not reach statistical significance, there was a noticeable trend toward increasing OHRQoL disparities with age, possibly reflecting evolving parental concerns as the systemic burden of NF1 intensifies over time. Taken together, these findings underline the importance of incorporating both general and oral health perspectives into the holistic management of NF1, with particular attention to the changing needs and perceptions of patients and families at different developmental stages. Integrating OHRQoL assessments into clinical practice could support early identification of emerging oral health concerns and foster age-sensitive, multidisciplinary care strategies aimed at improving both oral and overall quality of life in this vulnerable population.

This study has several limitations. NF1 patients were recruited solely from a single genetics clinic, likely representing milder cases, while those with potentially more severe symptoms followed in other departments were excluded. The broad age range may have obscured age-specific clinical features, particularly in younger children. Control subjects, drawn from a pediatric dental clinic, may not reflect the general population, possibly inflating oral health concerns in that group. Disease-specific NF1 severity or visibility scales were not used, as most participants exhibited mild symptoms; inclusion of more severe cases might have produced stronger findings. Given NF1’s progressive nature, cross-sectional design limits interpretation, highlighting the need for longitudinal studies. Finally, due to the syndrome’s psychosocial variability, repeated, systematic quality of life assessments are recommended.

as part of multidisciplinary care that should include pediatric dental professionals. On the other hand, using a 1:1 case–control ratio may have modestly limited statistical power compared with larger control groups, this design ensured close age/sex matching and high-quality data collection. Importantly, the study still provides novel, clinically relevant insights into the oral health and quality of life of children with NF1, supporting its value for guiding pediatric dental and multidisciplinary care. Despite these limitations, our study provides valuable preliminary insights into an underexplored aspect of NF1 care, offering an important foundation for future research and underscoring the potential benefits of incorporating oral health evaluations into the comprehensive management of children with NF1.

## Conclusions

Neurofibromatosis type 1 (NF1) is a complex genetic disorder with a wide spectrum of systemic and oral manifestations, many of which emerge during early developmental stages and can negatively impact both general and oral health–related quality of life. Our study contributes to the growing body of literature by providing detailed insights into the oral health status and its potential impact on quality of life in children with NF1, including the first evaluation of OHRQoL in this population. While our findings are largely consistent with previous studies regarding caries prevalence, oral soft tissue changes, and malocclusion rates, certain discrepancies were noted—likely reflecting methodological variations, the mild phenotype of our cohort, and the syndrome’s inherent heterogeneity. Importantly, despite relatively mild oral findings, children with NF1 reported significant impairment in general quality of life, particularly in physical functioning, underscoring the broader psychosocial burden of the condition.

These observations highlight the necessity of adopting an integrated, age-sensitive, and family-centered approach in the care of NF1 patients. Pediatric dentists, as part of the multidisciplinary team, can play a pivotal role not only in managing oral health issues but also in contributing to the early identification of NF1 through awareness of characteristic oral features. Notably, the case of the 7-year-old male patient in our cohort who presented with a central giant cell granuloma further illustrates how careful dental evaluation may facilitate timely referral for genetic assessment and definitive diagnosis. Strengthening interdisciplinary collaboration between dental, genetic, and medical professionals, alongside improving education on neurocutaneous syndromes within pediatric dentistry curricula, is essential to enhance patient care pathways. Finally, future prospective, multicenter, and longitudinal studies using standardized diagnostic criteria and quality of life assessments are warranted to better define the oral phenotype of NF1, refine management strategies, and support holistic, patient-centered care throughout the lifespan.

## Data Availability

The datasets generated and/or analysed during the current study are available from the corresponding author on reasonable request.

## Abbreviations

CALM: Café–au–lait macule
COHIP: Child Oral Health Impact Profile
CPQ: Child Perceptions Questionnaire
CPI: Community Periodontal Index
DMFT: Decayed, Missing, and Filled Teeth (permanent dentition)
dft: Decayed and Filled Teeth (primary dentition)
ECOHIS: Early Childhood Oral Health Impact Scale
EQ: 5D–5 L–EuroQol Five Dimensions Five Levels
GI: Gingival Index
HRQoL: Health–Related Quality of Life
KIDSCREEN: 10–KIDSCREEN–10 Index
NF1: Neurofibromatosis Type 1
NIH: National Institutes of Health
OHI: S–Simplified Oral Hygiene Index
OHRQoL: Oral Health–Related Quality of Life
PedsQL: Pediatric Quality of Life Inventory
PI: Plaque Index
POQL: Pediatric Oral Health–Related Quality of Life
QoL: Quality of Life
SPSS: Statistical Package for the Social Sciences
VAS: Visual Analogue Scale
